# Revealing the activation mechanism of autoinhibited RalF by integrated simulation and experimental approaches

**DOI:** 10.1038/s41598-021-89169-5

**Published:** 2021-05-12

**Authors:** Balint Dudas, David Perahia, Erika Balog

**Affiliations:** 1 Department of Biophysics and Radiation Biology, Semmelweis University, Budapest, Hungary; 2 Faculty of Information Technology and Bionics, Pázmány Péter Catholic University, Budapest, Hungary; 3 Laboratoire de Biologie et de Pharmacologie Appliquée, Ecole Normale Supérieure Paris-Saclay, Gif-sur-Yvette, France

**Keywords:** Computational biophysics, Molecular modelling, Molecular conformation

## Abstract

RalF is an Arf GEF from *Legionella pneumophilia,* the bacterium that causes severe pneumonia. In its crystal structure, RalF is in the autoinhibited form. A large-scale domain motion is expected to lift the autoinhibition, the mechanism of which is still unknown. Since RalF is activated in the presence of the membrane, its active structure and the structure of the RalF-Arf1 complex could not have been determined experimentally. On the simulation side, it has been proven that classical Molecular Dynamics (MD) alone is not efficient enough to map motions of such amplitude and determine the active conformation of RalF. In this article, using Molecular Dynamics with excited Normal Modes (MDeNM) combined with previous experimental findings we were able to determine the active RalF structure and the structure of the RalF-Arf1 complex in the presence of the membrane, bridging the gap between experiments and simulation.

## Introduction

*Legionella pneumophilia,* the bacterium that causes severe pneumonia (Legionnaire’s disease)^1^ is found both in many natural and artificial aquatic environments, such as cooling towers or air-conditioning and water systems of buildings. Recently, building closures caused by the Covid19 pandemic have drawn attention to the danger of Legionnaire’s disease^2^ indicating the necessary preventive measures to be taken.

To invade their host and avoid being destroyed, *Legionella pneumophilia* injects a large number of effectors—that alter membrane trafficking—into the target cell to establish a vacuole where it hides and replicates. One of these effector proteins is RalF, which is a guanine nucleotide exchange factor (GEF) of the ADP-ribosylation factor1 (Arf1)^3^, that diverts the function of Arf1 in the host. Arf1 belongs to the guanine nucleotide binding protein family of small GTPases controlling the membrane trafficking and organelle structure formation. Its function is regulated by the GDP-GTP cycle, being active in the GTP-bound and inactive in the GDP-bound form. As in small GTPases, the intrinsically very slow GDP/GTP exchange rate is catalyzed by two regulatory proteins: (i) the GTPase activating proteins (GAPs) that catalyze the GTP hydrolysis, resulting in the GDP-bound inactive conformation, and (ii) the guanine nucleotide exchange factors (GEFs) that catalyze the release of GDP, which is followed by GTP binding. RalF, being an Arf1-GEF, promotes the formation of the active GTP-bound Arf1. It has been shown that to propagate the signal transduction in the cell, both small GTPases, such as Arf1, and their regulators, in this case the invasive bacterial RalF, need to bind to the plasma membrane^4–7^.

RalF is composed of two domains, the human GEF homologue Sec7 (residues 1–190) and an additional Capping domain (residues 201–354), the two being connected by the Linker segment. The N-terminal Sec7 domain contains the Arf1 binding site where the catalysis of GDP to GTP exchange takes place (see Fig. S1). The crystal structure of RalF shows that the C-terminal Capping domain controls the exposure of the active site of RalF: it is in autoinhibited (closed) conformation in the cytosol, where the Arf1 binding site is covered by the Capping domain. Experimental results report enzyme activity in the presence of the membrane and find membrane-binding residues being identical to the Capping domain’s autoinhibitory motif^8^. To bind to the membrane, the autoinhibition of RalF has to be lifted, giving rise to an active site exposed to the solvent at the Sec7 domain and thus enabling the binding of Arf1. How this transition (closed to open) occurs and furthermore, the open, active structure of RalF have been unknown so far. The only available experimental structure is in a closed conformation in solution^9^; the membrane-bound open structure could not have been determined experimentally.

In this article, combining experimental findings with an efficient simulation method, we bridge the gap between experiments and classical MD simulation and determine the membrane-attached open RalF conformation and the all-atom structure of the membrane-attached RalF-Arf1 complex, also pointing out the essential residues that play a role both in opening and complex formation.

## Materials and methods

### RalF in solution

The starting crystallographic coordinates for RalF were taken from the Protein Data Bank, entry 1XSZ^9^ (Fig. S1). Since a large conformational change is expected when the autoinhibition is lifted, a rectangular box of TIP3 water molecules with 17 Å in all directions from the protein surface was generated with CHARMM-GUI^10,11^, and the NaCl concentration was set to 0.15 M. MDeNM simulations and analyzes were performed with CHARMM^12^ while MD simulations were carried out with NAMD^13^, both using the additive all-atom CHARMM force field C36^14^. The parameters of GDP were taken from our previous study^15^, and of myristoyl from the existing myristic acid parameters of CHARMM.

The solvated system was energy minimized with progressively decreasing harmonic restraints imposed to atomic positions: steepest descent was first used where the harmonic force constant was decreased every 100 steps adopting the values 50, 10, 1, and 0.1 kcal/mol/Å^2^, followed by 100 steps of unconstrained steepest descent minimization and 1000 steps of adopted basis Newton–Raphson.

Then the system was heated to and equilibrated at 300 K for 100 ps in an NVT ensemble followed by a 5 ns NPT equilibration run at 1 atm pressure. Langevin dynamics was used with a damping coefficient of 1 ps^−1^, a piston oscillation period of 50 fs, and a piston oscillation decay time of 25 fs. The integration time step was set to 1 fs during the 100 ps NVE and to 2 fs during the 5 ns NPT equilibrations.

For the energy calculations, the dielectric constant was set to 1. The particle mesh Ewald (PME) method was used to calculate the electrostatic interactions with a grid spacing of 1 Å or less having the order of 6; the real space summation was truncated at 12.0 Å and the width of Gaussian distribution was set to 0.34 Å^-1^. Van der Waals interactions were reduced to zero by ‘‘switch’’ truncation operating between 10.0 and 12.0 Å.

### MDeNM simulation of RalF in solution

**Figure 1:**
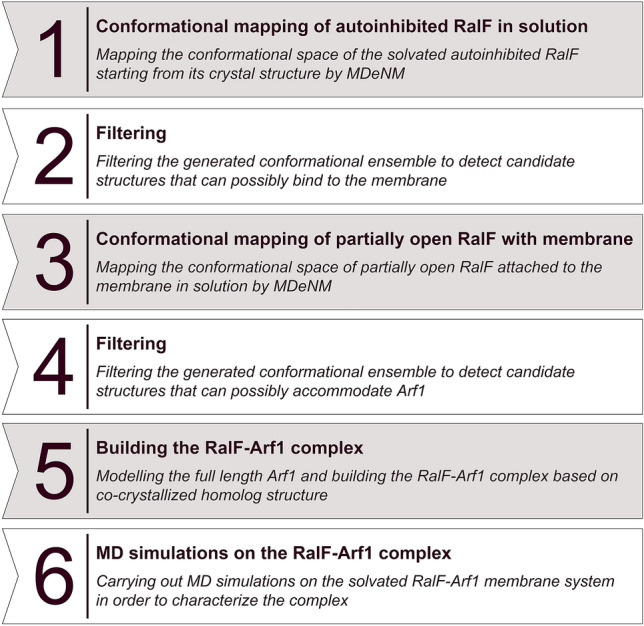
The steps of the modelling process starting from the autoinhibited, closed RalF and resulting in the relaxed, membrane-attached RalF-Arf1 complex.

**Figure 2:**
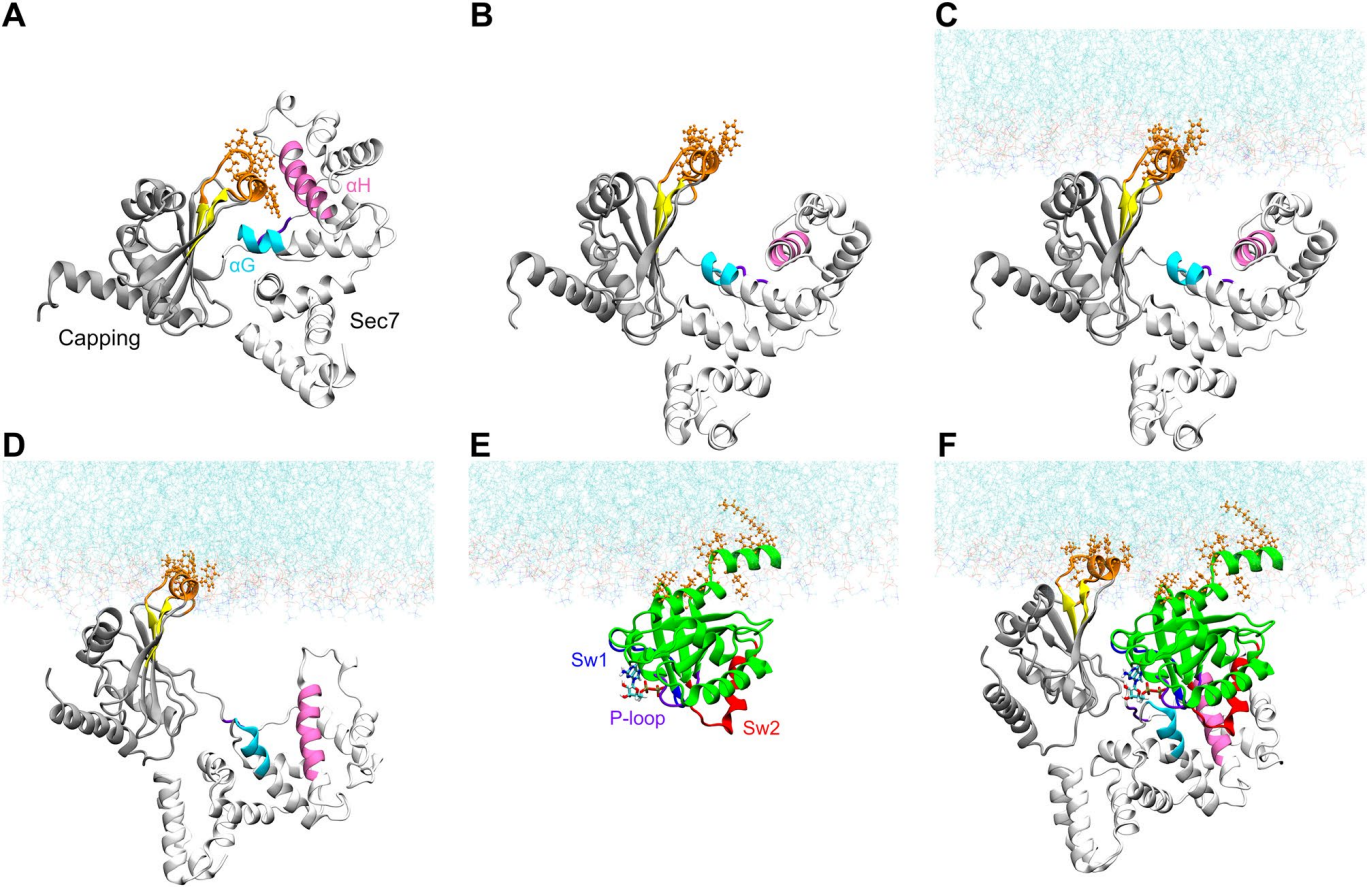
Structures from the different phases of the modelling process: (**A**) autoinhibited, closed RalF; (**B**) partially open RalF retrieved after the filtering of the first conformational mapping step; (**C**) partially open RalF obtained previously anchored to the membrane; (**D**) open RalF attached to the membrane retrieved by MDeNM starting from the anchored structure shown in (**C**); (**E**) the modeled membrane-anchored, myristoylated, full-length Arf1; and (**F**) the relaxed, membrane-attached RalF-Arf1 complex. RalF Sec7 is represented in white denoting the Arf1 binding site by cyan (αG) and mauve (αH). The Capping domain is represented in grey. Arf1 depicted in green, switch1 in blue, switch2 in red, and P-loop in purple. The GDP nucleotide is in licorice representation, the membrane-binding residues are in orange.

Starting from the equilibrated closed RalF structure in solution, the MDeNM^16^ approach was used to thoroughly map its conformational surface (step 1 in Figs. 1 and 2A). MDeNM combines normal mode analysis (NMA) and molecular dynamics (MD) to investigate significant proteins’ conformational changes. MDeNM performs several simultaneous MD simulations in which different randomized linear combinations of the most relevant low frequency normal modes are kinetically activated.

The normal modes used in the MDeNM simulations were calculated on the final structure of the 5 ns equilibration MD run. The structure was first energy minimized using the steepest descent method with the harmonic force constant of atomic restraining potentials decreasing every 500 steps, adopting the values 10, 1, 0.1, and 0 kcal/mol/Å^2^ followed by adopted basis Newton–Raphson till a tolerance of RMS energy gradient of 10^–5^ kcal/mol/Å was reached. The normal modes of the energy minimized structure were then calculated using the VIBRAN module of CHARMM. For the MDeNM calculations, the 10 lowest frequency normal modes were taken based on their RMSF contribution and were applied to the final structure of equilibration run.

Then, during MD simulations, the system was excited along the randomized linear combinations of the 10 modes. Kinetic excitations were performed every 2000 steps in the form of velocity increments equivalent to an overall temperature rise of 5 K of the system, yielding a set of conformations for a given replica. The inserted energy dissipates rapidly during the 4 ps relaxation period, which is why the kinetic excitations are renewed. For the MD runs the same parameters were used as described previously.

For the thorough mapping of the conformational space, the new combinations of modes were systematically compared to the previously accepted excitation directions. They were only kept if the RMSDs between 1 Å displaced structures along these directions were greater than 1.15 Å. In total, 264 different combinatorial directions were accepted, for each of which 16 excitations were applied, resulting in 4224 conformations.

Based on the experimental results of Folly-Klan et al.^8^, the conformations on the generated ensemble were systematically tested to fulfill the following four criteria (step 2 in Fig. 1, for structural details see Fig. S3): (i) Y326 remains in contact with the Sec7 domain, (ii) the membrane-binding residues (W251, F254, F255, I282, F283, K285, W286, L287) foreseen experimentally^8^ become uncovered, (iii) the Arf1 binding site remains undistorted—this was monitored by the distance between residues E103 and F137, and (iv) the Sec7 and the Capping domain retain their native fold. This last criterion was necessary because the release of a strong autoinhibitory interaction between the Capping and Sec7 domains following a large kinetic excitation by MDeNM could perturb their structure. Indeed, the energy injected along certain combinations of the low frequency normal modes can lead to some non-native conformations, making this filtering step necessary.

### Selection of RalF conformations that can bind to the membrane

The filtering process aimed to identify conformations with their expected membrane-binding residues exposed to the solvent so that the lipid bilayer, which has been reported to play a pivotal role in RalF activation^9^, can be included in the next simulation steps. Figure 2B shows an example of such a partially open structure. As shown in the orange CPK representation, the residues forming the twin helices, W251, F254, F255, I282, F283, and K285, that are buried in the closed conformation (Fig. 2A) became exposed to the solvent, enabling them to be attached to the membrane (Fig. 2C).

### RalF associated to the membrane

The OPM server^17^ was used to determine the orientation of the partially open RalF conformation to the membrane. The OPM results on the partially open conformation agree with the results reported by Folly-Klan et al.^8^, the membrane-binding residues being identified as F254, I282, F283, K285 and F288.

Using the orientation given by OPM, the partially open RalF conformation attached to the membrane was built by CHARMM-GUI, which is represented on Fig. 2C. As the figure shows, this intermediate structure can already attach to the membrane but is not open enough to accommodate the Arf1 molecule. Thus, a second MDeNM conformational sampling was necessary, starting from the partially open conformation, with the lipid bilayer included (step 3 in Fig. 1).

### RalF MDeNM simulation in the presence of membrane

With the orientation described above, the membrane bound partially open, TIP3 solvated system was generated by CHARMM-GUI^18^. The NaCl concentration was set at 0.15 M. As the bacterial RalF is injected in the human cell, a model membrane of DOPC/DOPS 80:20 mol ratio was constructed following the work of Jang et al.^19^. The system was energy minimized, heated, and equilibrated with the same protocol as described above. The final structure of the protein after the equilibration was then energy minimized for calculating its normal modes. A second MDeNM was then applied to the whole system with the same parameters as described previously.

The generated conformations were again systematically filtered (step 4 in Fig. 1) to fulfill the experimental criteria listed above supplemented with a fifth condition: (v) the Arf1 binding site (residues 100–103 denoted by cyan and 130–145 denoted by mauve in Fig. 2) becomes exposed to the solvent, so that RalF can bind Arf1. This was enforced by a distance criterion between the residues F137 and F255 (See Fig. S3). Figure 2D represents an example of such an open structure.

### Selection of RalF conformations that can accommodate Arf1

The RalF-Arf1 complex was built using the previously chosen open RalF structures and the human Arf1 structure, taken from the PDB entry 1R8S^20^. Since 1R8S contains Arf1 co-crystallized with the Sec7 domain of Arno^21^, we constructed the RalF-Arf1 complex directly from the 1R8S entry by superposing the Sec7 helices of the Arf1 binding site of RalF and Arno structures (residues 87–97, 110–122, 129–148, 177–190). No steric clashes were found in the presented RalF-Arf1 complex.

### Building the model with full-length Arf1

Since the crystal structure of the human Arf1 (1R8S) used in the construction of the RalF-Arf1 complex does not contain the mobile N-terminal helix of Arf1 (resid 1–17), the myristoylated N-terminal helix was taken from the full length NMR structure of the yeast Arf1, PDBID: 2KSQ^22^, choosing the N-terminus orientation for which the membrane-binding residues are coplanar with the membrane-binding residues of RalF (step 5 in Fig. 1). The sequence identity of the N-terminal 1–17 amino acids of human and yeast Arf1 is 56%; the sequence similarity is 91% (for the full length the identity is 75% while the similarity is 93%). The following residues of the yeast Arf1 N-terminal structure (residues 1–17) were then mutated back to correspond to the human form: L3N, F4I, A5F, S6A, K7N, S10K, N11G, N15K (for structural characterization of Arf1 see Fig. S2). To verify the stability and characterize the complex (Fig. 2F) further MD simulations of the full-length RalF-Arf1 complex were performed.

### MD of the membrane-attached RalF-Arf1 complex

As a final step, after the RalF-Arf1 complex was built, the complex was oriented by OPM, and the oriented structure was attached to the DOPC/DOPS 80:20 bilayer and solvated in a TIP3 water box by CHARMM-GUI with a 0.15 M NaCl concentration. The system was energy minimized, heated and equilibrated, then three independent 50 ns long MD simulations were carried out starting from the same equilibrated complex with different initial velocity distributions. The parameters of the MD simulations were the same as those described above.

### MD simulations on the autoinhibited RalF

To compare the conformations reached by MDeNM with the conformations accessible by conventional MD simulations, we performed three independent 200 ns long MD simulations with different initial velocity distributions from the closed, auto-inhibited RalF structure—the initial conformation also used by the first MDeNM simulations in solvent. The parameters for the 200 ns MD runs were identical to those of the previously performed MD and MDeNM simulations.

For convenience, the performed simulations are also summarized in Table S1 of Supp Inf.

### Interaction energies

To elucidate the behavior of the membrane-attached RalF-Arf1 complex and the differences between inactive and active RalF, the interaction energies were analyzed. They were calculated as a sum of pairwise non-bonded electrostatic and van der Waals energy contributions. For the energy calculations CHARMM was used with the same force field parameters as for the MD and MDeNM simulations detailed above. The interactions were calculated by considering the atoms of residue pairs between the two domains of RalF, between RalF and Arf1, and between the proteins and the lipid bilayer; they were evaluated at the amino acid level. The energy values reported are statistical averages of the given interaction energy acting between two residues, calculated among the structures retrieved from the MD simulations.

## Results and discussion

As no active RalF structure has been resolved experimentally, we started our simulations from the autoinhibited crystal structure of RalF (for structural details see Fig. S1). Applying multiple MDeNM, filtering, and classical MD steps (for a stepwise overview, see Figs. 1 and 2) enabled us to determine the RalF-Arf1 complex associated with the membrane.

### Comparison of the inactive and active RalF

In the process of autoinhibition release by large-scale interdomain rearrangements, existing interdomain interactions disappear, new interactions are formed, and some interactions are maintained, playing a central role in the RalF activation. These rearrangements ensure that RalF effectively binds the membrane and Arf1.

### Rearrangement of intramolecular interactions, hinge point identification

**Figure 3:**
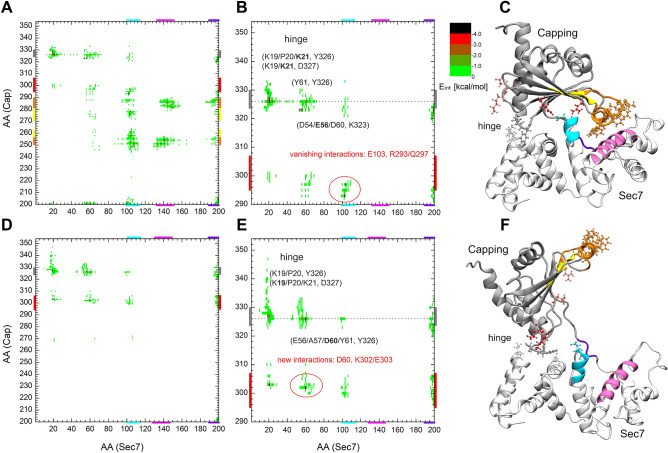
Interaction energy map between the residues of the Sec7 and the Capping domain of RalF in its autoinhibited, closed (**A**) and active, open state (**D**). Part (**B**) and (**E**) are enlarged views of A and D respectively, focusing on the hinge site. Part (**C**) of the figure shows the closed crystal structure and, (**F**) a modelled, open conformation of RalF. Structural regions of both domains color-coded along the axes of A and D are mapped with identical coloring onto the **C** and **F** part of the figure.

To detect the presence or lack of interaction, the interaction energy between the residues of the two domains was calculated. Figure 3 shows the interaction energy map obtained in the autoinhibited crystal structure (A, D) and the open conformation (B, E) where binding of Arf1 is sterically possible.

In the autoinhibited form (Fig. 3A–C) amino acids forming the Arf1 binding site of the Sec7 domain (colored in cyan and mauve on the figures) and the Linker region (colored in violet) show interactions with the membrane-binding residues (colored in orange) and with part of the β-core of the Capping domain (colored in yellow). These interactions stabilize the autoinhibited form of RalF. Comparison of the residue pair interaction maps between the closed (Fig. 3A) and open (Fig. 3D) conformations shows how the above-mentioned interactions disappear when the Capping domain is displaced from the Sec7 domain. In particular, the interactions between the membrane-binding residues W251, L252, F254, F255, I282, F283, and L287 of the Capping domain^8^ (orange; also see Fig. S4 for a magnified view), vanish with the residues constituting the Arf1 binding site E103, I107, Y133, F137, M141, T144, and I151 of the Sec7 domain and residues L194 and F196 of the Linker region upon RalF activation.

Figure 3B,E shows the enlarged view of the interaction maps A and D for residues 290–354 of the Capping domain. The comparison of the enlarged closed and open RalF interaction maps clearly shows the loss of the interaction of the catalytically active glutamate E103 (denoted by cyan CPK in Fig. 3C) with the residues R293 and Q297 (represented by red CPK on Fig. 3C and shaded red CPK in Fig. 3F, for magnified view see Fig. S4). E103 becomes freely accessible in the open form, as can be seen in Fig. 3F (represented by cyan CPK).

The new interactions formed in the open conformation are of residues K302 and E303 (denoted in shaded red in Fig. 3C and red in Fig. 3F) with residues D60, E66, and K21 respectively. These interactions contribute to the stabilization of the active conformation and thus to the facilitation of Arf1 binding.

In the closed and open conformations, an extended interaction of the Capping domain residues Y326 and D327 with K19, P20, K21 and D60, Y61 of the Sec7 domain (denoted by grey CPKs in Fig. 3C,F and in Fig. S4) can be seen, suggesting that these residues act as a *hinge* in the opening/closing motion of RalF.

### Comparison between the performance of MD and MDeNM in RalF opening

To compare the performance of MDeNM and conventional MD in conformational search of RalF, three 200 ns long MD simulations were performed starting from the same closed, autoinhibited RalF structure as the one considered for the MDeNM simulations.

**Figure 4:**
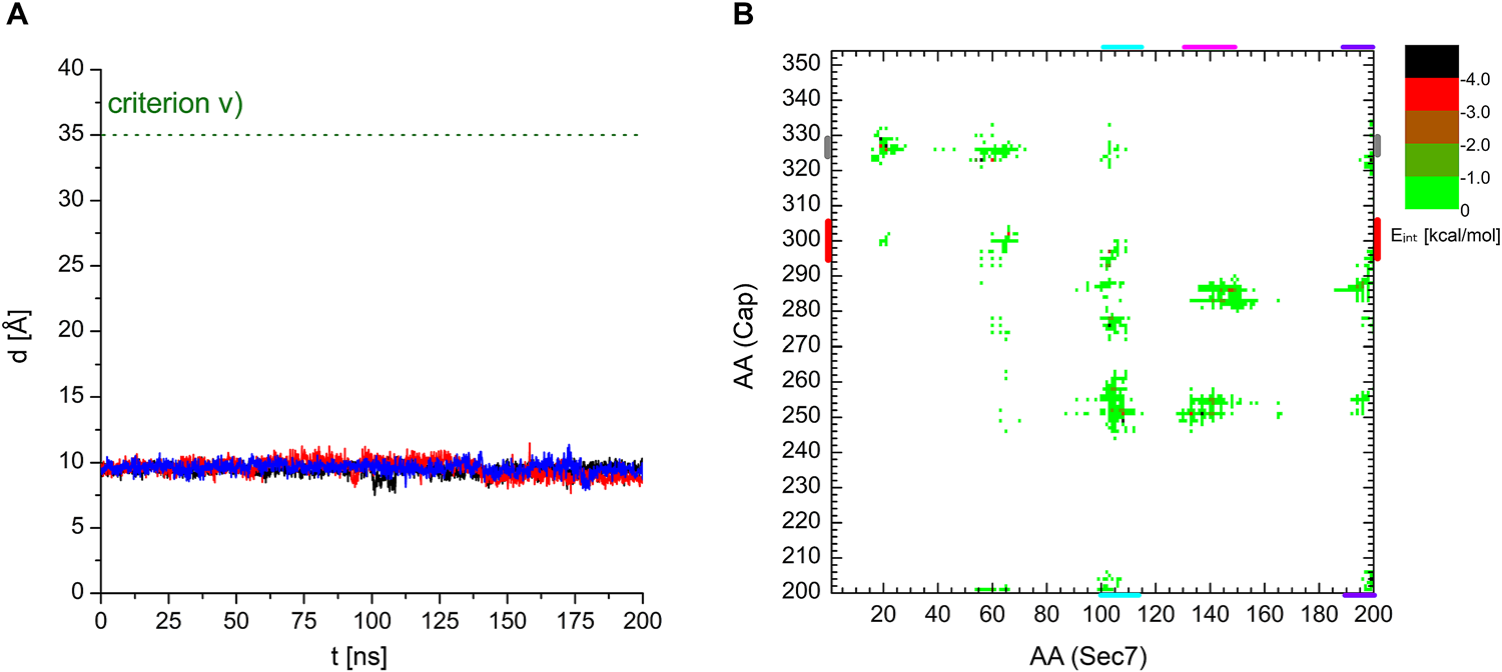
(**A**) Evolution of the filtering *parameter v)*, the distance between the C_α_ atoms of the Sec7 domain residue F137 and Capping domain residue F255, during the three concurrent 200 ns MD runs starting from the closed, autoinhibited RalF conformation. (**B**) Interaction energy map between the residues of the Sec7 and the Capping domain, calculated on the conformations retrieved from the three MD runs.

Among other quantities monitored during the modeling process described above, *parameter v)*, the distance between the residue F137 of the Sec7 domain and the residue F255 of the Capping domain follows the openness of the different conformations. Based on the diameter of Arf1 and its orientation with respect to the Sec7 domain, a lower estimate of *parameter v)* for conformations capable of accommodating Arf1 was determined as 35 Å, hence *criterion v)* was set accordingly. Figure 4A shows that the F137-F255 distance is practically constant, varying around 10 Å, in the three MD simulations, not showing any tendency of RalF opening with classical 200 ns MD. For comparison, this distance is 9.2 Å in the closed crystal structure of RalF and 47.2 ± 1.1 Å during the MD simulations of the stable RalF-Arf1 complex.

Furthermore, for comparison with MDeNM results, interactions between the residues of Sec7 and the Capping domain were calculated over the three MD simulations and are shown in Fig. 4B. The interaction map of the RalF MD generated structures is almost identical to that of the autoinhibited starting structure of RalF presented in Fig. 3A, showing all the interactions existing between Sec7 and the Capping domain in the crystal structure. This also confirms, in agreement with the visual inspection of the MD trajectories, that RalF remains autoinhibited throughout the MD simulations.

### Interactions in the RalF-Arf1 complex

After constructing the RalF-Arf1 complex, as described in the Material and Methods section, three independent 50 ns MD simulations were performed. A measure of the stability of the complex was the time evolution of the openness of RalF (followed by *criterion v*) through the simulations (see Fig. S5), showing a stationary behavior with an average of 47.2 Å, as mentioned before.

**Figure 5:**
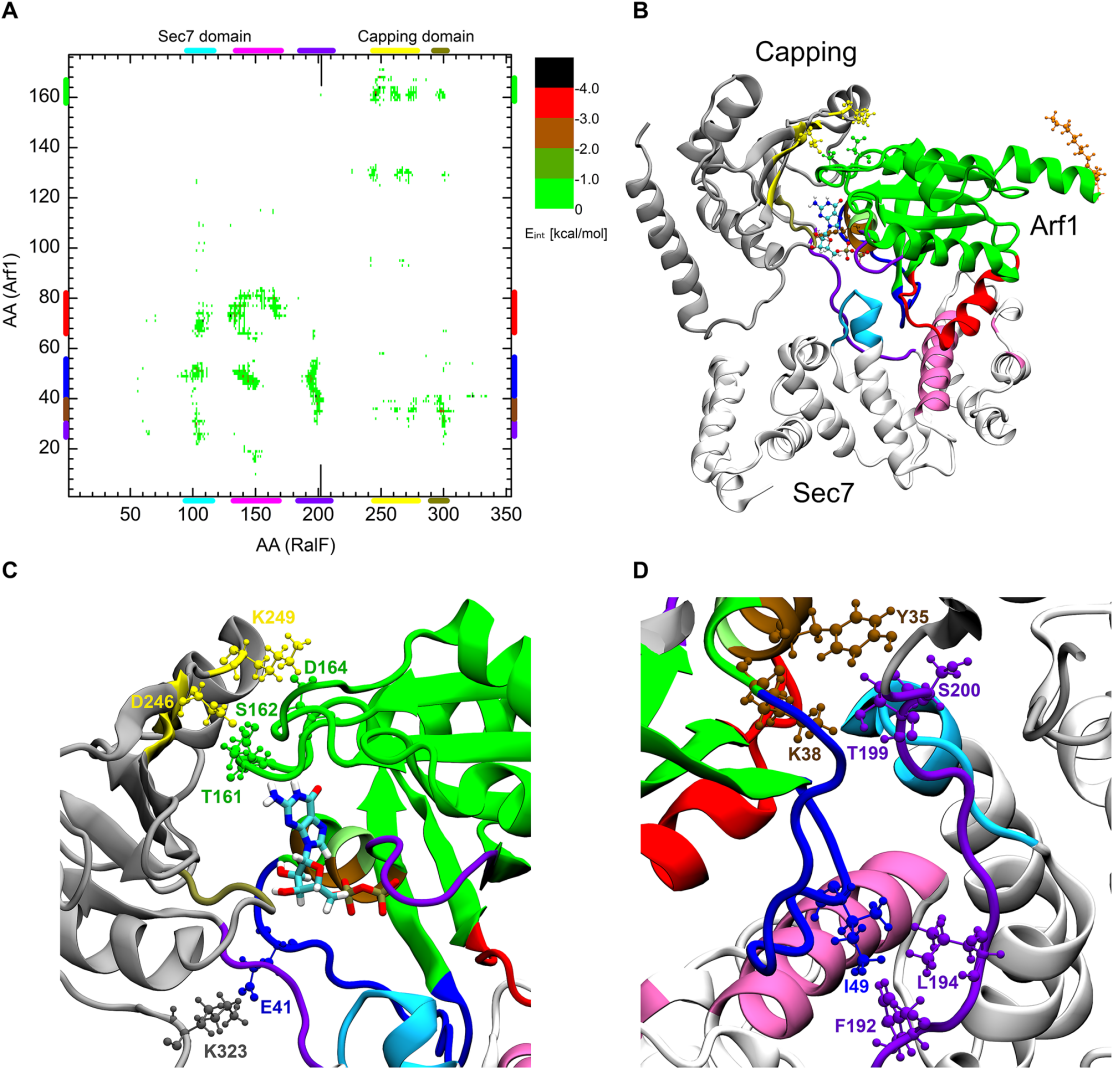
(**A**) the interaction energy map between the residues of Arf1 and RalF. (**B**) 3D structure of the RalF-Arf1 complex. Magnified views of the RalF-Arf1 Capping domain and RalF-Arf1 Linker region are shown in part (**C**) and (**D**) of the figure respectively.

The interaction energies between RalF and Arf1 residue pairs within the complex are shown in Fig. 5A.

The map, which is in agreement with interactions seen in crystal structures of other GEFs Sec7 domains complexes with Arf^23–26^, in addition to showing the interactions of RalFSec7-Arf1, reveals the existence of a previously unknown extensive interaction pattern between the RalF Linker region and the RalF Capping domain with Arf1.

### Sec7 domain-Arf1 interactions

Since the RalF-Arf1 complex was built by overlapping the Sec7 domain of open RalF onto the Sec7 domain of the co-crystallized complex of Arf1-Sec7 of Arno, switch1 (residues 45–53, denoted in blue) and switch2 (residues 68–84, denoted in red) of Arf1 were expected to maintain their orientation with respect to the predicted active site of RalF similar to what can be found in homologous Sec7-Arf1 complexes (entries with PDBID: 1R8Q^20^, 6FAE^27^, 1RE0^24^).

Indeed, throughout the three independent MD simulations of the complex, the hydrophobic clamps^20^ of switch1 and switch2 sustained close spatial proximity to the hydrophobic groove^23^ of the Sec7 active site (see Fig. S6A) of RalF. Statistics on the shortest distances between these interacting residues during the three independent MD simulations are presented in Table S2 of Supp Inf. Preservation of these interactions throughout the MD simulations also supports the stability of the complex.

Furthermore, besides the hydrophobic clamps, two residue pairs D130^RalF^/L73^Arf1^ and M108^Ralf^/G71^Arf1^ form hydrogen bonds, stabilizing the complex (see Fig. S6B).

### Capping domain- and Linker region-Arf1 interactions

In contrast to the prior knowledge of Sec7 domain-Arf1 interactions from the co-crystalized structures, there was no information about the Capping domain-Arf1 interactions. Our results indicate that Arf1 interacts with both the Capping domain and the Linker region.

Within the Capping domain residues at the edge of the RalF beta strands: β3, β4, and β5 (denoted by yellow in Fig. 5) interact with the Arf1 loop between β6 and αF (residues 160–164). The strongly interacting residue pairs which hold this part of the complex together are D246^RalF^/T161^Arf1^-S162^Arf1^ forming double hydrogen bond; K249^RalF^/D164^Arf1^ and K323^RalF^/E41^Arf1^ of switch1 forming a salt bridge interaction which are represented on Fig. 5C.

As part A of the figure shows, the RalF Linker region (residues 192–200, denoted by light violet) also plays a role in the stabilization of Arf1 in open RalF. At the C-terminus of the Linker a hydrogen bond is formed between residues S200^Ralf^/Y35^Arf1^ and T199/K38^Arf1^ of αB of Arf1; while at its N-terminal hydrophobic residues F192^RalF^ and L194^RalF^, which are involved in Capping domain interactions in the inactive RalF, form hydrogen bonds with I49^Arf1^ of switch1 (Fig. 5D).

Analyzing the complex from the nucleotide point of view, Table S2 of Supp Inf shows that during the MD simulations residues Q272^RalF^, Q300^RalF^, and T301^RalF^ stayed in close proximity to the GDP (bound to Arf1). Notably Q300 interacts strongly with the ribose ring of the nucleotide in the relaxed complex indicating its potential involvement in the enzymatic activity of RalF.

### Interactions with the membrane

**Figure 6:**
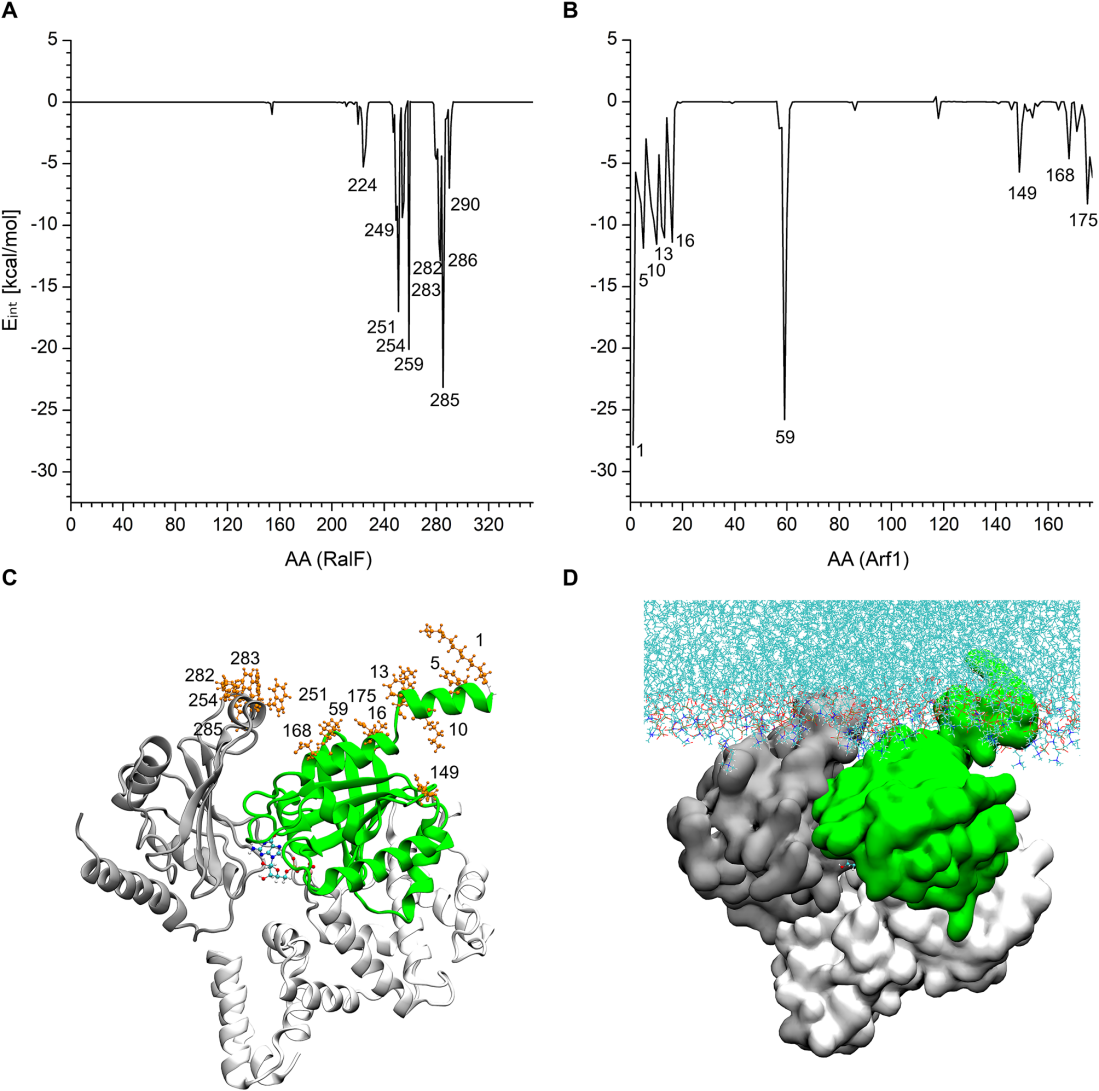
Interaction energy of the residues of (**A**) RalF and (**B**) Arf1 with the membrane. 3D structure of the RalF-Arf1 complex in (**C**) cartoon and (**D**) surface representation. The most interacting residues are represented in orange CPK. The RalF Sec7 is shown in white, the Capping domain in grey, and the Arf1 in green.

Figure 6 presents the interactions of the RalF-Arf1 complex with the membrane throughout the three MD runs. In agreement with previous findings^8^, the vicinity of RalF amino acids W251, F254, and K285 are embedded in the membrane (Fig. 6A).

Figure 6B shows that (i) the N-terminal myristoylated amphipathic helix (αA) anchors Arf1 into the membrane, which is in agreement with previous NMR results^22,28^; (ii) another strong interaction of Arf1 with the membrane is of the residue K59, which lies at the tip of the interswitch region and it has also been predicted to have a role in localizing Arf1 to the membrane^5^. Our results also show that (iii) the C-terminus of Arf1 also secures the positioning of the protein on the membrane.

The stability of the membrane-anchored RalF-Arf1 complex was further confirmed by the time percentage of the membrane contact calculations of the above listed residues, showing constant contact with the membrane throughout the MD simulations (Table S3).

## Conclusions

In this theoretical–experimental integrated approach the use of the MDeNM method was essential for generating the necessary large scale movements. This method, which uses the low frequency normal mode directions and their combinations as guiding directions to promote large scale motions, has been shown to be a powerful approach to studying functional and allosteric motions of proteins in solvent^15,16,29^, and interacting with membranes^30,31^. In our recent study we showed that while in the case of the Kir channel, the gating mechanism for the passage of the potassium ion exceeds the timescale of seconds, MDeNM required simulations of 45 ns to reveal the large structural changes involved^31^. In the case of capturing the open RalF conformation, the present study shows that MDeNM simulations of less than 20 ns were sufficient.

In this article the combined use of (i) the MDeNM for conformational sampling, (ii) the experimental data to filter the conformational ensemble generated by this method, and (iii) a co-crystallized homologous Sec7-Arf1 complex structure available in the PDB, it was possible to lift the autoinhibition of RalF and generate a full-length all-atom RalF-Arf1-membrane complex. We identified in details the opening mechanism, the hinge point of autoinhibited Ralf, and the essential residues that stabilize the Ralf-Arf1 complex.

While neither experimental techniques nor classical MD simulations were sufficient to model the lift of the auto-inhibition of RalF and determine the structure of its active form, let alone the structure of the RalF-Arf1 complex in the presence of the membrane, by using an efficient sampling method, this work demonstrates the usefulness of an integrated experimental-simulation approach that can be used to build and study structures that gain a function in a biological process.

## Supplementary Information

Supplementary Information.
