# Supplementary material for: Revealing the activation mechanism of autoinhibited RalF by integrated simulation and experimental approaches
Source: Sci Rep. 2021 May 12;11:10059. doi: 10.1038/s41598-021-89169-5 (PMC8115643; doi:10.1038/s41598-021-89169-5)
Supplement: Supplementary file 1 — Supplementary Information. [file 41598_2021_89169_MOESM1_ESM.docx]

**Supplementary Information for**

**Revealing the activation mechanism of autoinhibited RalF by integrated simulation and experimental approaches**

Balint Dudas^1,2^, David Perahia^3^, Erika Balog^1*^

*Corresponding author:

balog.erika[@med.semmelweis-univ.hu](mailto:xxxxx@xxxx.xxx)

**
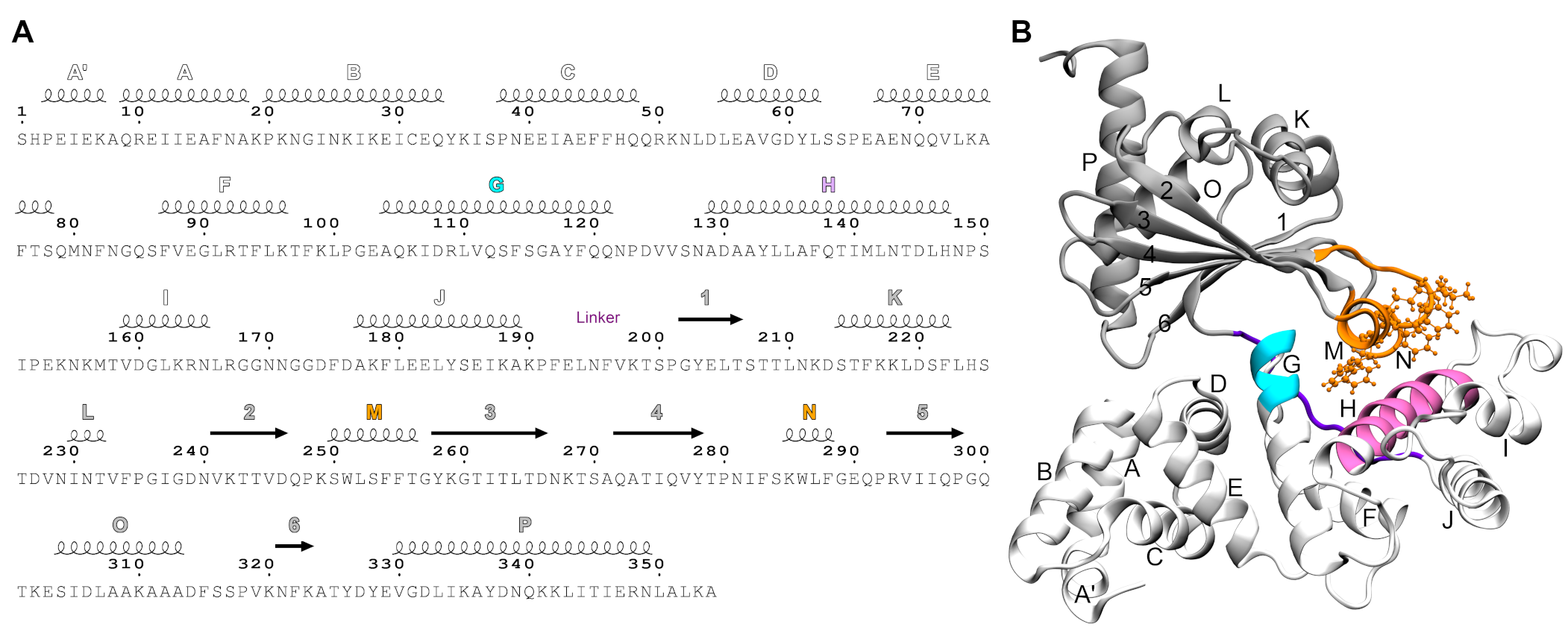
**

**Fig. S1.** The sequence (A) and structure (B) of RalF. The secondary structural elements are marked on both part of the figure. Sec7 domain is denoted by white, indicating the Arf1 binding site by cyan (αG) and mauve (αH), while the Capping domain is shown in grey indicating the predicted membrane-binding segment by orange. The color coding used on the A panel is mapped on the three-dimensional structure.

**
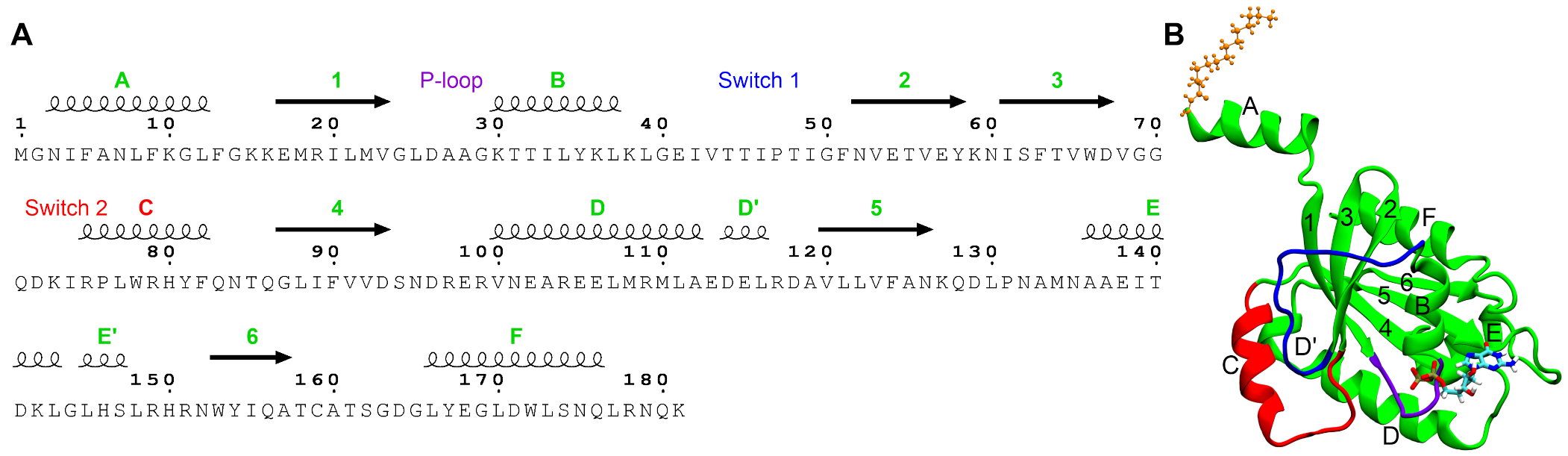
**

**Fig. S2.** The sequence (A) and structure (B) of Arf1. The secondary structural elements are marked on both part of the figure. Switch1 is represented in blue, switch2 in red, and the P-loop in purple. The color coding used on the A part of the figure is mapped on the three-dimensional structure.

**
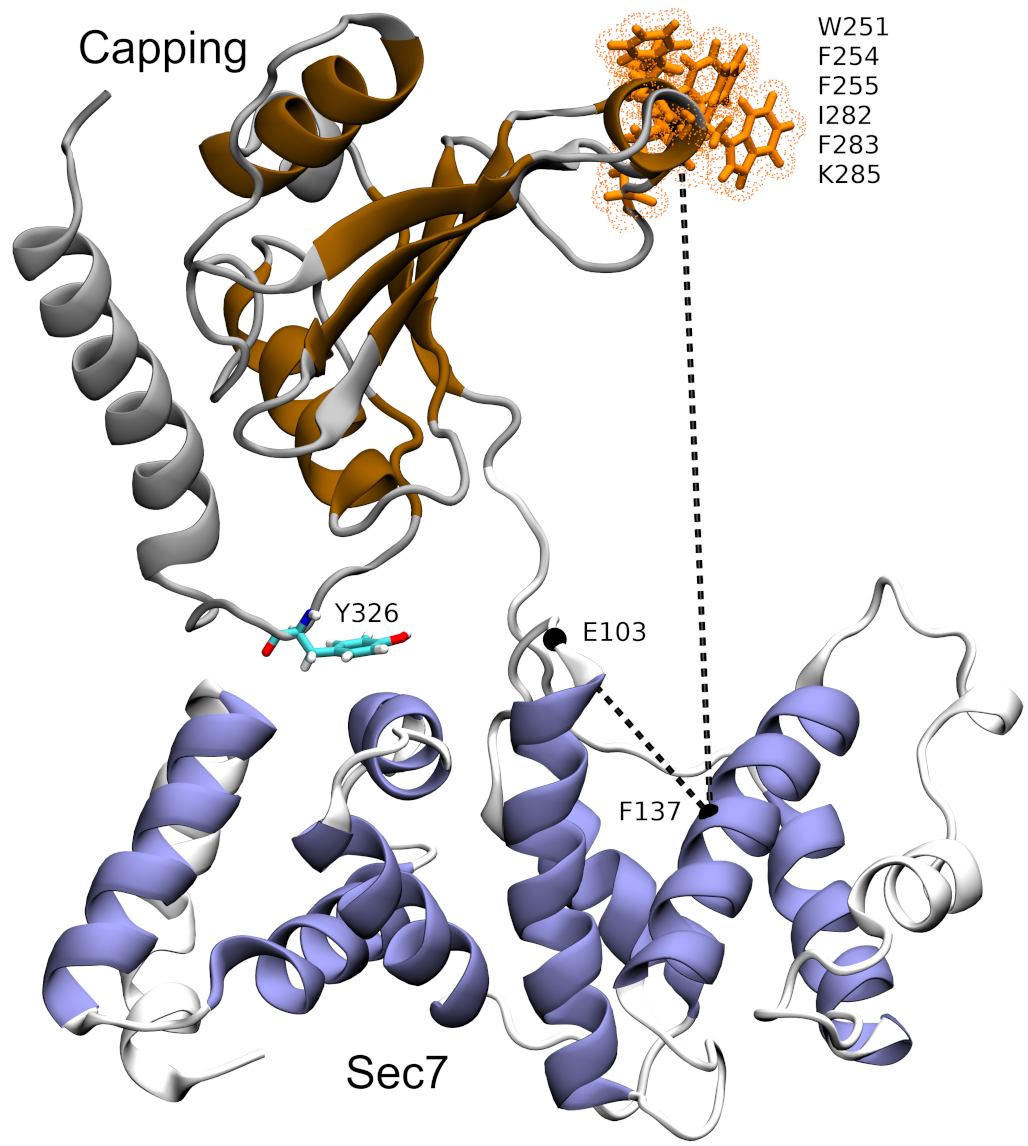
A** **B**

| **Monitored Parameter** | **Description** |
| --- | --- |
| i | RMSD calculated within rigid secondary structural elements independently for the two domains |
|  |  |
| ii | Shortest distance between the Capping domain residue Y326 and the Sec7 domain |
|  |  |
| iii | Solvent Accessible Surface (SAS) calculated for the predicted membrane binding residues |
|  |  |
| iv | Distance between the C_α_ atoms of the Sec7 residues F137 and E103 |
|  |  |
| v | Distance between the C_α_ atoms of the Sec7 domain residue F137 and Capping domain residue F255 |
|  |  |

**Fig. S3.** Description of the monitored parameters for filtering (A) and their graphical representation on the open conformation of RalF (B).

| **System** | **Simulation Type** | **Simulated Time** | **Comment** |
| --- | --- | --- | --- |
| RalF in solvent | MD Equilibration | 5 ns |  |
|  | MD Production | 3 x 200 ns (= 600 ns) | *"Dead-End"* |
|  | MDeNM | 264 replicas x  16 excitations x 4 ps  (= 16.9 ns) | *Results filtered on criteria: i,ii,iii,iv* |
| RalF + membrane | MD Equilibration | 20 ns |  |
|  | MDeNM | 264 replicas x  16 excitations x 4 ps  (= 16.9 ns) | *Results filtered on criteria: i,ii,iii,iv,v* |
| RalF + Arf1 +  + membrane | MD Production | 3 x 50 ns (= 150 ns) |  |

**Table S1.** Summary of the simulation protocol. The filtering criteria are detailed in Fig S3.

**
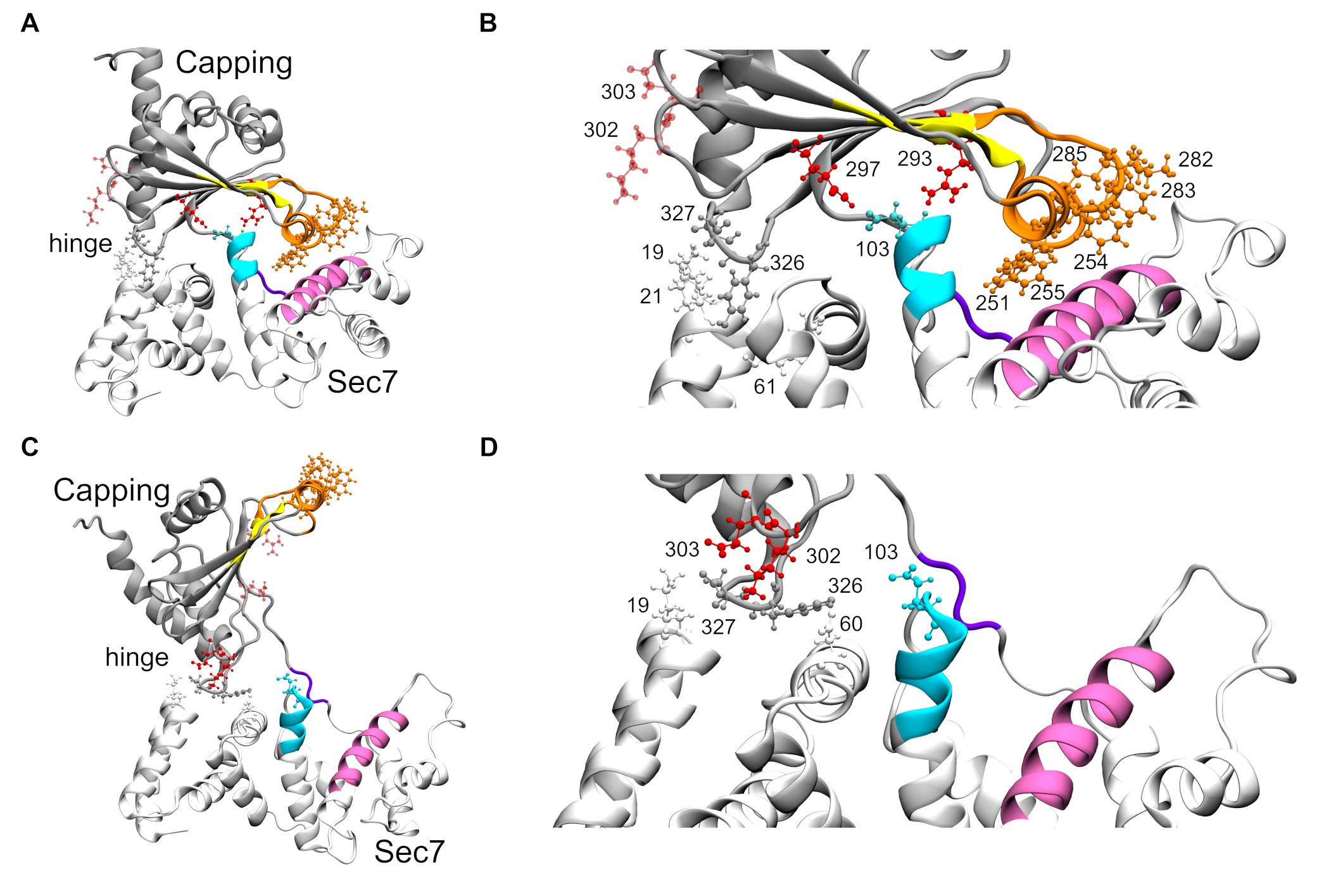
**

**Fig. S4.**  The closed crystal structure of Ralf (A; B the magnified view) and a modelled, open conformation (C; D the magnified view). The membrane binding site is represented by orange, the hinge by grey CPKs. The Arf1 binding sites is shown by cyan and mauve.


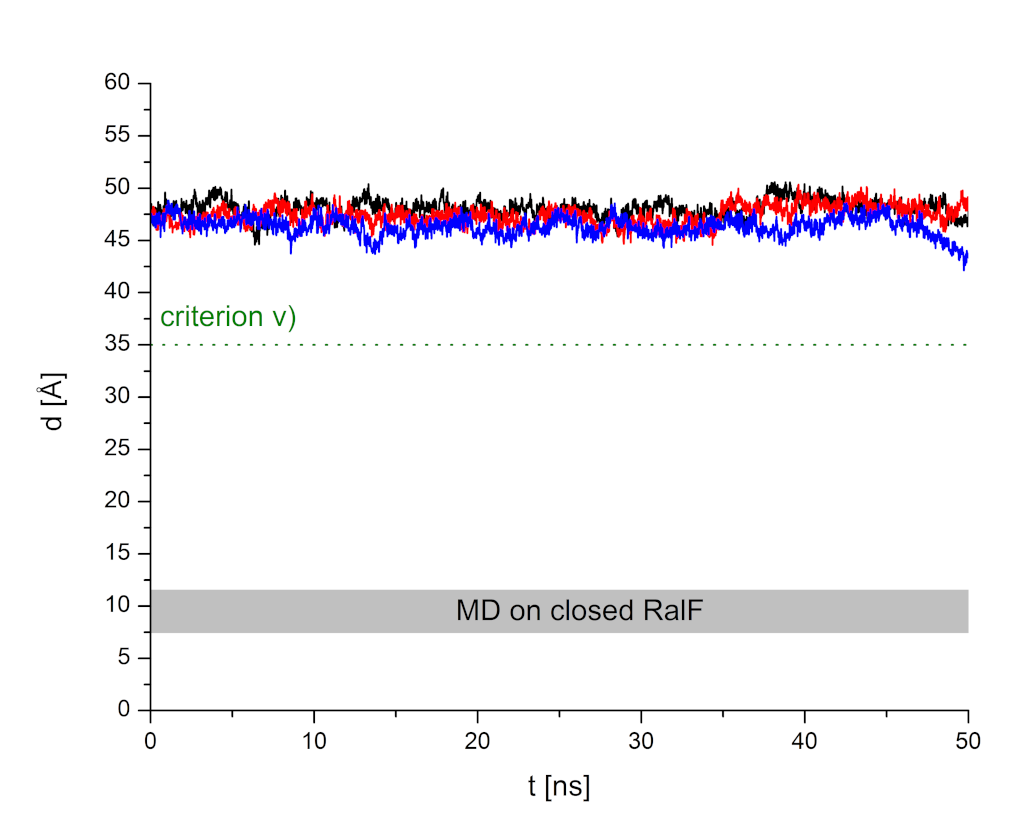


**Fig. S5.** Time evolution of the filtering *parameter v)*. The distance between the C_α_ atoms of the Sec7 domain residue F137 and Capping domain residue F255 during the three concurrent 50 ns MD runs (denoted by black, red and blue) staring from the equilibrated RalF-Arf1 complex; for comparison, the explored range of the closed RalF MD and the *criterion v* used for the filtering is presented.

**
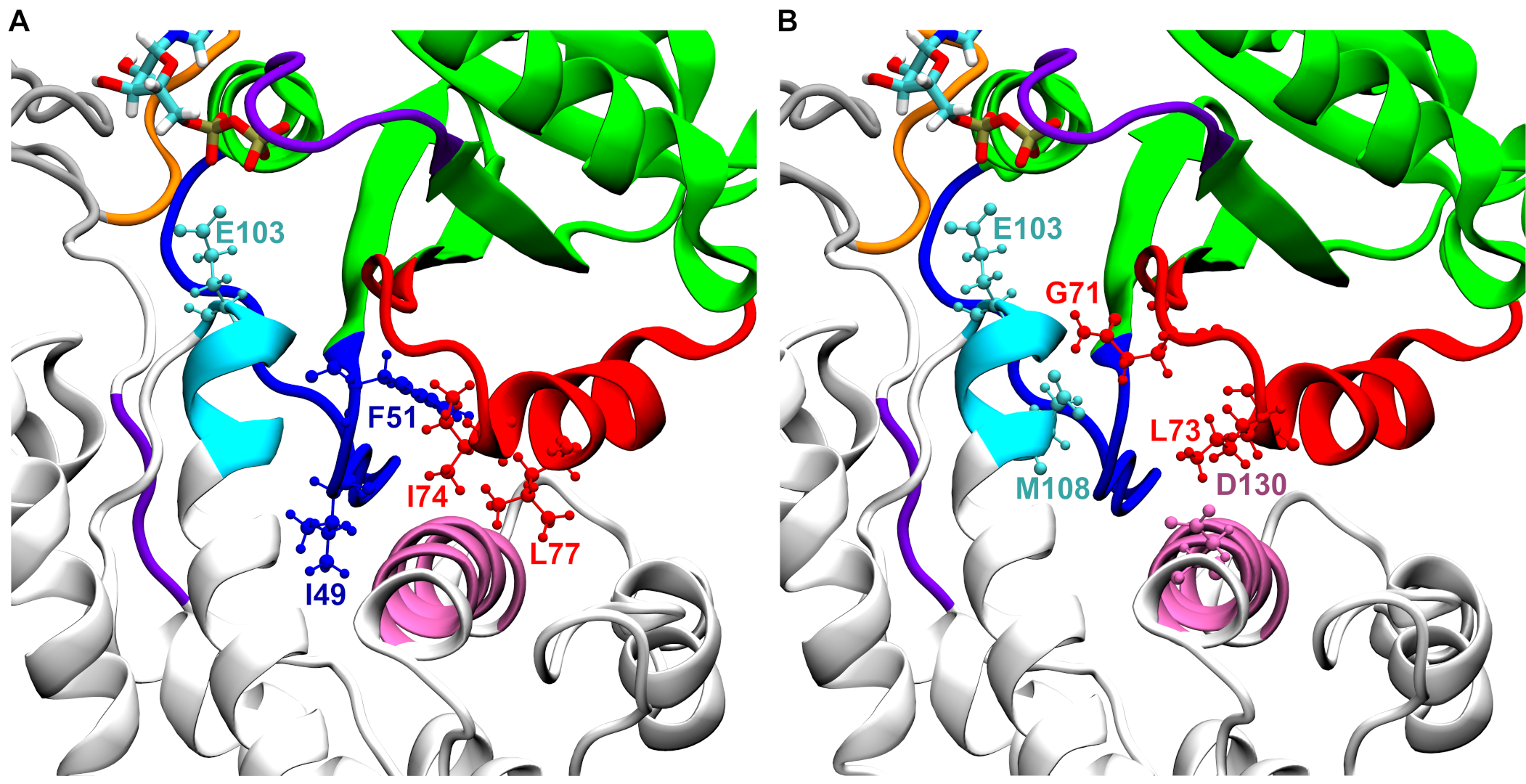
**

**Fig. S6.** Magnified views of Arf1(green)-RalF (white) complex. The Sec7 active site of RalF is denoted by cyan (α7) and mauve (α8), while switch1 and switch2 of Arf1 are colored by blue and red, respectively. A. The hydrophobic clamps of Arf1 (I49, F51, I74, L77) shown by CPK as well as the catalytic glutamate (E103, cyan). B. Strong hydrogen bonding residue pairs of RalF Sec7 domain -Arf1.

|  | **AA_RalF** | **AA_Arf1** | **AVG** [Å] | **STD** [Å] |
| --- | --- | --- | --- | --- |
| **Sec7-Arf1** | **I140** | **I49** | 2.3 | 0.2 |
|  | **F192** |  | 2.5 | 0.4 |
|  | **L194** |  | 2.7 | 0.5 |
|  | **A104** | **F51** | 2.6 | 0.3 |
|  | **I107** |  | 2.8 | 0.4 |
|  | **F137** |  | 3.0 | 0.6 |
|  | **M141** |  | 2.7 | 0.4 |
|  | **L134** | **I74** | 3.2 | 0.8 |
|  | **F137** |  | 2.6 | 0.3 |
|  | **L134** | **L77** | 2.6 | 0.5 |
|  | **F137** |  | 2.6 | 0.4 |
|  | **M141** |  | 2.7 | 0.4 |
|  | **D130** | **L73** | 2.4 | 0.9 |
|  | **M108** | **G71** | 3.0 | 0.9 |
| **Capping-Arf1** | **D246** | **T161** | 2.1 | 0.9 |
|  |  | **S162** | 2.7 | 1.0 |
|  | **K249** | **D164** | 4.3 | 2.3 |
|  | **K323** | **E41** | 4.0 | 2.1 |
| **Linker-Arf1** | **F192** | **I49** | 2.5 | 0.4 |
|  | **L194** |  | 2.7 | 0.5 |
|  | **T199** | **K38** | 2.8 | 0.8 |
|  | **S200** | **Y35** | 3.3 | 1.2 |
| **RalF-GDP** | **Q272** | **GDP** | 3.7 | 1.1 |
|  | **Q300** |  | 2.5 | 0.4 |
|  | **T301** |  | 2.2 | 0.3 |

**Table S2.** Average and standard deviation of the shortest residue-residue distances of the RalF-Arf1 complex during the three independent MD simulations. Residues I49, F51 (of switch1) and I74, L77 (of switch2) form the hydrophobic clamps of Arf1.

| **RalF_AA** | **%** |  | **Arf1_AA** | **%** |
| --- | --- | --- | --- | --- |
| H224 | 74 |  | Myr1 | 100 |
| K249 | 71 |  | F5 | 100 |
| W251 | 100 |  | K10 | 76 |
| F254 | 100 |  | F13 | 100 |
| K259 | 85 |  | K16 | 70 |
| I282 | 100 |  | K59 | 97 |
| F283 | 100 |  | R149 | 35 |
| K285 | 100 |  | E168 | 58 |
| W286 | 100 |  | N175 | 95 |
| E290 | 77 |  |  |  |

**Table S3.** Time percentage of membrane contact for the enlisted residues during the 3x50 ns MD of the RalF-Arf1 complex. The contact is defined by the shortest distance between the heavy atoms of the residues and the membrane being less than 3 Å.
